# Mechanism and biomass association of glucuronoyl esterase: an α/β hydrolase with potential in biomass conversion

**DOI:** 10.1038/s41467-022-28938-w

**Published:** 2022-03-18

**Authors:** Zhiyou Zong, Scott Mazurkewich, Caroline S. Pereira, Haohao Fu, Wensheng Cai, Xueguang Shao, Munir S. Skaf, Johan Larsbrink, Leila Lo Leggio

**Affiliations:** 1 Department of Chemistry, University of Copenhagen, DK-2100 Copenhagen, Denmark; 2 College of Chemistry, Research Center for Analytical Sciences, Tianjin Key Laboratory of Biosensing and Molecular Recognition, State Key Laboratory of Medicinal Chemical Biology, Nankai University, 300071 Tianjin, P. R. China; 3 Wallenberg Wood Science Center, Division of Industrial Biotechnology, Department of Biology and Biological Engineering, Chalmers University of Technology, SE-412 96 Gothenburg, Sweden; 4 Institute of Chemistry and Center for Computing in Engineering and Sciences, University of Campinas – Unicamp, Campinas, SP 13084-862 Brazil

**Keywords:** Enzyme mechanisms, Hydrolases, X-ray crystallography, Biocatalysis, Computational chemistry

## Abstract

Glucuronoyl esterases (GEs) are α/β serine hydrolases and a relatively new addition in the toolbox to reduce the recalcitrance of lignocellulose, the biggest obstacle in cost-effective utilization of this important renewable resource. While biochemical and structural characterization of GEs have progressed greatly recently, there have yet been no mechanistic studies shedding light onto the rate-limiting steps relevant for biomass conversion. The bacterial GE *Ot*CE15A possesses a classical yet distinctive catalytic machinery, with easily identifiable catalytic Ser/His completed by two acidic residues (Glu and Asp) rather than one as in the classical triad, and an Arg side chain participating in the oxyanion hole. By QM/MM calculations, we identified deacylation as the decisive step in catalysis, and quantified the role of Asp, Glu and Arg, showing the latter to be particularly important. The results agree well with experimental and structural data. We further calculated the free-energy barrier of post-catalysis dissociation from a complex natural substrate, suggesting that in industrial settings non-catalytic processes may constitute the rate-limiting step, and pointing to future directions for enzyme engineering in biomass utilization.

## Abstract (web summary)

Zong and coworkers combine computational and experimental methods to decipher in detail the mechanism of action of glucuronoyl esterases, enzymes with significant biotechnological potential for decoupling lignin from polysaccharides in biomass.

## Introduction

Lignocellulose is an important resource, for instance as feedstock for production of clean and renewable energy carriers, such as bioethanol, and furthermore both lignin and polysaccharides are potential sources of building blocks for materials and fine chemicals, alternative to the ones derived from fossil resources^1,2^. Lignocellulose is, however, an extremely complex and heterogeneous substrate, and is highly recalcitrant to enzymatic and chemical deconstruction^3^. A substantial obstacle is the chemical linkages between lignin and carbohydrates, the so-called lignin carbohydrate complex (LCC)^4^. Lignin is often an obstacle in lignocellulose processing and its removal has been regarded as the most expensive step for producing ethanol from plant biomass in industry, suggesting that recalcitrance is the uppermost cost factor^5,6^. Decreasing lignocellulosic recalcitrance and enhancing its degradability are, therefore, of paramount importance to lower the cost of bioconversion. Furthermore, lignin itself is a valuable biomass component with many possible applications. Thus, improving separation of polysaccharide and lignin fractions will additionally favor lignin valorization^2^.

Glucuronoyl esterases (GEs, EC number 3.1.1.117 at https://www.brenda-enzymes.org/) from carbohydrate esterase family 15 (CE15) in the carbohydrate-active enzymes database (CAZy; www.cazy.org)^7^ have been reported to attack small model substrates since their first discovery^8^ and are believed to attack lignin–carbohydrate ester bonds in LCCs^9^. To date, several fungal and bacterial CE15 enzyme structures have been determined^9–15^. Their biochemical features have been characterized using model substrates, such as alkyl and aryl alcohols of varying complexity ester linked to glucuronic acid (GlcA) or 4-*O*-methylated GlcA^9–11,16–28^, and in some cases with more natural ones, such as extracted LCC fractions^29–32^. Native LCC ester bonds are found between GlcA moieties branching from xylan polysaccharides and lignin moieties in the plant cell wall. Their exact nature has been heavily debated, with much discussion as to whether benzyl (α) esters and/or γ-esters are represented in native lignin, or if different species can be produced during extraction and pre-treatment^33–35^. However, it has been clearly shown that plant cell wall recalcitrance is greatly reduced in *Arabidopsis* mutants devoid of GlcA, further compounding the importance of ester linkages to GlcA as a main interface between xylan and lignin^36^.

Regardless of the nature of native esters in the plant LCC, synergy between GEs and depolymerizing enzymes in biomass degradation is well documented. In 2016, the first example for GE–cellulase synergy in degrading lignocellulosic biomass was reported by d’Errico et al.^19^. More sugars were found to be released from corn fiber in the presence of the fungal GE from *Cerrena unicolor*, compared to using commercial cellulases alone. In the study of Arnling Bååth et al., direct evidence of cleavage of native lignin–carbohydrate ester bonds was provided using the GE originating from *Acremonium alcalophilum*^29^. Furthermore, the bioconversion of ball-milled corncob by the commercial enzyme cocktail Ultraflo^®^ was substantially increased when supplemented with different GE enzymes indicating an important role for the enzymes in synergistically enhancing biomass degradation^9^. Recent work has also shown that GEs can synergistically act together with α-glucuronidases to boost release of 4-*O*-methylated GlcA from native LCC fractions^32^.

CE15 GEs belong to the α/β hydrolase (ABH) superfamily and possess a Ser–His–Glu/Asp catalytic triad like many well-studied serine-type hydrolases^9,10,37–39^. In several fungal GEs, which were the first characterized members of the family^12,13^, the position of the catalytic acid is at the end of β-strand 6 (β6), rather than at the end of β7 as in classical ABHs^37^ (here referred to as canonical position). The canonical position of the acid has however been identified in several bacterial GEs studied since^11,14^. ABHs, comprising several industrially and biomedically important enzymes, have been extensively investigated. However, detailed mechanistic studies on GEs have not yet been carried out. Furthermore, the GE from *Opitutus terrae* (*Ot*CE15A) is unusual, possessing an acidic residue at the end of β6 in addition to the classical triad, in other words acidic residues both at the end of β6 (non-canonical position) and β7 (canonical position). In addition to the fundamental interest for the function of this important and widespread enzyme class, a better understanding of the GE mechanism is also of high societal importance. Further studies on variations of the classical triad are important in inspiring the engineering of enzymes and synthetic enzyme mimics in a variety of contexts^40^. A deeper understanding of the possible rate-determining step for GEs degrading their complex natural substrate will also be extremely valuable in protein engineering efforts devoted to improving their industrial potential.

As a classical ABH, the reaction catalyzed by *Ot*CE15A was assumed to proceed through a covalent intermediate preceded by an acylation and followed by a deacylation step, both going through a tetrahedral transition state. We then explored this canonical carboxyl esterase mechanism^41^ through quantum mechanics/molecular mechanics (QM/MM) simulations. A free energy method was additionally employed to investigate dissociation to infer the rate-limiting step for GE degradation of lignocellulose. To support the in silico results, we also performed additional biochemical and structural studies. The present work provides new fundamental understanding of catalytic triad variations in serine hydrolases and will be of great use for further development of these industrially important enzymes.

## Results

### Initial choice of a minimal active site for mechanistic studies

**Fig. 1:**
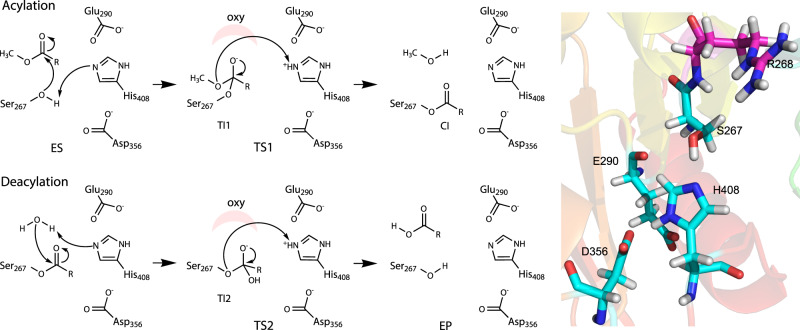
Catalytic mechanism corresponding to the hydrolysis of a glucuronic acid methyl ester (with the sugar moiety shown as R) by *Ot*CE15A. ES and EP denote non-covalent enzyme–substrate and enzyme–product complexes, respectively. TS1 and TS2 denote transition states in acylation and deacylation, respectively, and CI represents the covalent intermediate. TI1 and TI2 denote the tetrahedral intermediates in acylation and deacylation, respectively. Oxy represents the oxyanion hole. Glu290 and Asp356 correspond to the non-canonical and canonical positions, respectively, for the acidic residue in the ABH triad, both present in *Ot*CE15A. A 3D view of the active site is presented on the right (PDB 6SYR).

The catalytic site of ABHs, including GEs, is commonly constituted by residues Ser–His–Glu/Asp in a catalytic triad — the essential catalytic functional unit^9,10,12,42^. As for other ABHs, the nucleophilic Ser and general acid–base His are expected to be directly involved in the catalytic reaction (Fig. 1)^43,44^, and accordingly the S267A variant was severely impaired with a large decrease in turnover rate (*k*_cat_)^10^. The reaction is presumed to proceed through a covalent intermediate (CI), which has been experimentally demonstrated for the *Ot*CE15A H408A variant.

Although the acidic residues in ABH catalytic triads are not involved in the catalytic reaction directly, they are reported to stabilize the transition states, for example in acetylcholine esterase, by stabilizing the histidine charge during the reaction^43,44^. As described above, in contrast to other studied GEs, *Ot*CE15A features acidic residues at both the canonical and non-canonical positions, i.e., Glu290 and Asp356^10,15^. The formation of the CI (acylation step) and breakdown of the CI to release the final product (deacylation) both go through formation of a tetrahedral transition state with a charge on the oxygen stabilized by the so-called oxyanion hole^42^. In many ABHs, this is formed solely by backbone nitrogen atoms from the enzyme, but for bacterial GEs, the side chain guanidinium moiety of the Arg immediately following the Ser nucleophile (Arg268 in *Ot*CE15A) has been proposed to play this role^10,11^. As a starting point in this investigation, we focus on Ser267, His408 and Glu290 (the non-canonical position and the position found in the first characterized GEs) as the minimal set of active site residues needed to investigate the catalytic mechanism of *Ot*CE15A, and, as such, the side chains of these residues and a small model substrate were included in the QM region considered (SHE).

### Acylation

**Fig. 2:**
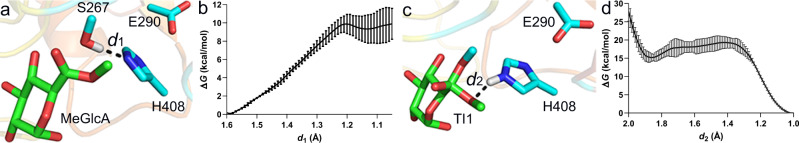
Processes of acylation with the minimal active site assembly. **a** Initial structure of *Ot*CE15A and MeGlcA constructed based on in silico esterification of GlcA complex of *Ot*CE15A (based on PDB 6SYR). **c** Relevant TI1 state to describe the reaction in acylation. **b**, **d** Corresponding relevant free-energy profiles generated from QM/MM simulations. Carbon atoms of MeGlcA and the catalytic triad, i.e., Ser267, Glu290, and His408 in the QM area are shown in green and cyan, respectively. For clarity, some hydrogen atoms in the QM area, the counterions and water molecules are not shown, here and in the forthcoming figures. There are a total of 48 atoms in the QM area, and the charge is −1. *d*_1_ denotes the distance between the hydroxyl proton of Ser267 and Nε of His408. *d*_2_ denotes the distance between the proton of the Nε of His408 and O of the ester in MeGlcA. Data are presented as mean values ± the standard error inferred from three independent runs. Source data for **b**, **d** are provided as a Source Data file.

As shown in Fig. 2a, a GlcA methyl ester (MeGlcA) was constructed based on the complex of *Ot*CE15A and GlcA obtained in our previous work (PDB 6SYR)^10^, and used as the initial structure to investigate the acylation reaction. The transition coordinates (TCs) characterizing the reaction pathways in acetylcholine esterases have been investigated by Zhang et al.^43^ and Nemukhin et al.^44^ in detail, using a combined coordinates approach. A single TC has been adopted by Yan et al.^45^ and Zong et al.^46^ to investigate the mechanism of proton transfer in the glycosylation reaction of the *Trichoderma reesei* Cel7A cellulase successfully. In the present work, on the assumption that the catalytic reaction starts from proton transfer, we employed two individual distances as the TCs to investigate the two proton transfer processes involved in the acylation and deacylation steps. Two factors were taken into account. Firstly, although a single TC, such as *d*_1_ in Fig. 2a, was used to describe the first proton transfer process, another important process—nucleophilic attack, which is highly coupled with the first process, was found to be well sampled in our well-tempered variant of meta-eABF (WTM-eABF) simulation (as discussed below), indicating that using the individual distance as the TC is justified. Secondly, the free-energy barrier of each process in acylation and deacylation can be obtained by this approach, helping us find out the rate-limiting steps in the whole reaction and in each step.

Here, the distance between the hydroxyl proton of Ser267 and Nε in His408, *d*_1_, was used as the TC to describe the first proton transfer process, along which the potential of mean force (PMF) describing this process was determined over three independent runs. In our simulations, the proton transfer was found to be accompanied by the nucleophilic attack (Supplementary Movie 1 and Supplementary Fig. 1a). As depicted in Fig. 2b, the free-energy barrier against this first process is ~10 kcal/mol. Furthermore, three distances, i.e., *d*_1_, Ser267 nucleophilic O—carbonyl C in MeGlcA, and methanol group O—carbonyl C in MeGlcA were analyzed to monitor the processes of proton transfer and nucleophilic attack (Supplementary Fig. 1a). The result indicates that proton transfer and nucleophilic attack occur simultaneously to form the TI1. In the next step of acylation, the proton on Nε His408 donated by Ser267 will finally transfer to the O atom of the methanol group in MeGlcA. The distance between His408 Nε and O in MeGlcA, *d*_2_, was employed to describe this process (Fig. 2c). As depicted in Fig. 2d, there is no more than 4 kcal/mol barrier for the proton transferring to create CI and leaving group (LG) (Supplementary Movie 2). Corresponding distances were analyzed (Supplementary Fig. 1b), showing that the LG and CI form successfully. Overall, the first process, i.e., the proton transfer from Ser267 to His408 is the key process for acylation in energetic terms.

### Priming for deacylation

**Fig. 3:**
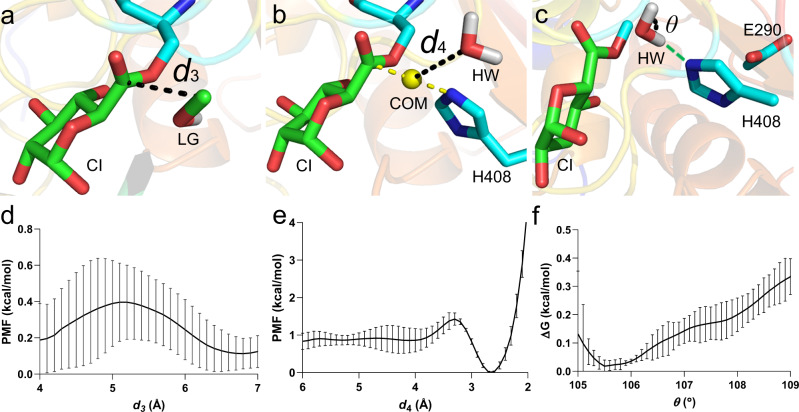
Processes before deacylation with the minimal active site assembly. **a**–**c** Initial structure of CI with **a** the LG in the catalytic site, **b** the water molecule (HW) near the catalytic site, and **c** HW now moved into the catalytic site (hydrogen bond formed with Nε of His408 is shown in green dotted line). **d**–**f** Corresponding PMFs calculated along reaction coordinates *d*_3_, *d*_4_, and *θ*, respectively. *d*_3_ denotes the distance between the C of CI and C in LG. *d*_4_ denotes the distance between the HW O atom and the yellow sphere, i.e., the center of mass (COM) of C in CI and Nε in His408. *θ* denotes the bond angle in HW. There are a total 45 atoms in the QM area for the angle-change calculation, and the charge is −1. Data are presented as mean values ± the standard error inferred from three independent runs. Source data for **d**–**f** are provided as a Source Data file.

As illustrated in Fig. 3a, a CI complex in silico was obtained from the trajectory of the third process in acylation. We have previously obtained an experimental CI structure for a H408A variant of *Ot*CE15A (PDB 6SZ4)^10^, aligning well with our computationally obtained structure (Supplementary Fig. 2). After the acylation reaction, the LG will leave the active site, creating sufficient space for a hydrolytic water (HW) molecule to attack the CI. The *d*_3_ coordinate, which measures the distance between LG C and CI C atoms, was, therefore, employed to describe this process of methanol group leaving the active site (Fig. 3a), along which the PMF was determined. Figure 3d indicates that the LG leaves the catalytic site with a negligible free energy transfer barrier.

After the methanol group leaves, the HW molecule will undergo two processes: (i) approach, i.e., the HW enters the active site region from the LG side, arrives at the catalytic position and forms a hydrogen bond with Nε of His408; (ii) angle change, i.e., its bond angle changes from approximately 105° to 109°^42^. In the crystal structure of CI (PDB 6SZ4), a structural water (Wat 704 in the PDB file) was located at the LG side (Supplementary Fig. 2). In our computational simulations, the water molecule nearest to the one in the crystal structure was thus selected and used for the following calculation. The PMF describing the approach process by the TC *d*_4_, i.e., the distance between the water O atom and the center of mass (COM) of CI C and Nε in His408 (Fig. 3b), was determined. As shown in Fig. 3e, the HW molecule was found to approach the catalytic region at a 0.6 kcal/mol energetic cost, and stabilizes the complex, mainly owing to the hydrogen bond formed with Nε of His408 (Fig. 3c).

After analyzing the simulation trajectory for the approach step, the structure corresponding to the minimum of the PMF in Fig. 3e was used as the starting model in the following QM/MM calculations for the angle change process (Fig. 3c). Figure 3f indicates that the HW molecule surmounts ~0.3 kcal/mol barrier to complete its angle change to 109°. In view of the marginal free-energy costs against the three processes of priming the active site for deacylation, we suggest that once the CI is formed, the enzyme will expel the LG and prepare the HW molecule quickly, getting ready for the next step—deacylation.

### Deacylation reaction

**Fig. 4:**
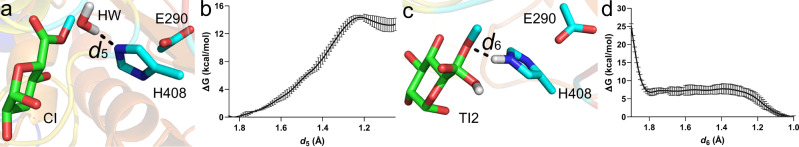
Processes of deacylation with the minimal active site assembly. **a**, **c** Initial structure of CI with (**a**) the HW and (**c**) the relevant TI2 state to describe the deacylation reaction; **b**, **d** Relevant free-energy profiles generated from QM/MM simulations. There are 45 atoms in the QM area, and the total charge is −1. *d*_5_ denotes the distance between the proton of HW molecule and Nε in His408. *d*_6_ denotes the distance between the proton of Nε His408 and O in CI. Data are presented as mean values ± the standard error inferred from three independent runs. Source data for **b**, **d** are provided as a Source Data file.

As depicted in Fig. 4a, the complex between the CI and HW, obtained from the aforementioned trajectory of QM/MM simulation, was employed as the initial structure to investigate the deacylation reaction. Here, *d*_5_, namely the distance between the HW proton–Nε in His408, was employed to describe the initial proton transfer, along which the PMF was determined. In our simulations, the processes of initial proton transfer from the water molecule and the nucleophilic attack of HW on CI were found to be concerted (Supplementary Movie 3). As shown in Fig. 4b, the barrier against the first proton transfer is 14.4 kcal/mol, which is higher than for acylation and indicates that deacylation is the rate-limiting step, as previously indicated by similar *k*_cat_ of *Ot*CE15A against a variety of substrates with different LGs^10^. The TC *d*_6_, denoting the distance between the proton of Nε His408 and O in CI was used to reveal the process of the final proton transfer (Fig. 4c). As illustrated in Fig. 4d, the determined PMF indicates that the energy is very favorable for this process (Supplementary Movie 4). In deacylation, the initial proton transfer from HW to His408 was thus considered as the dominant process.

### Finding the optimal QM region by including additional residues

**Fig. 5:**
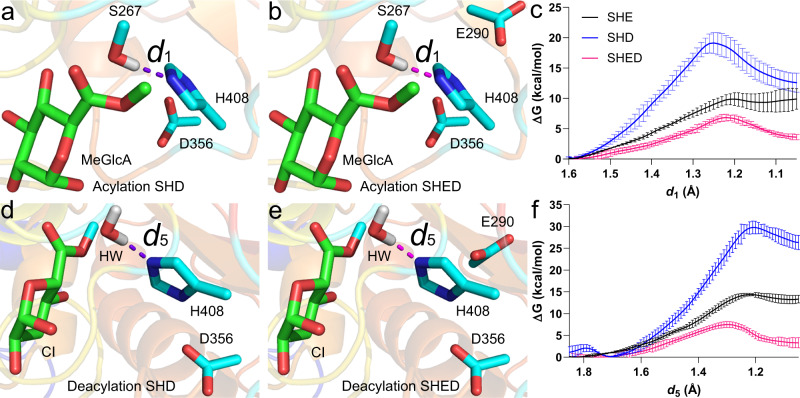
Initial proton transfers in the acylation and deacylation steps with the optimal SHED QM region. **a**–**c** Acylation step. **d**–**f** Deacylation step. In (**a**, **d**), QM areas of SHD include only one acidic residue for the calculations of acylation and deacylation, respectively. In (**b**) and (**e**), QM areas of SHED comprise two acidic residues for the calculations of acylation and deacylation, respectively. (**c**, **f**) show the relevant free-energy profiles generated from QM/MM simulations. The corresponding results of SHE (black lines) are shown for reference. Data are presented as mean values ± the standard error inferred from three independent runs. Source data for **c**, **f** are provided as a Source Data file.

Since *Ot*CE15A has two acidic residues in the active site, we also investigated Ser–His–Asp (SHD) and Ser–His–Glu–Asp (SHED) in addition to the Ser–His–Glu catalytic assembly (investigated above, SHE), to determine how important their inclusion in the QM region is to the calculated barriers. As shown in Fig. 5a, d, each QM area of SHD/SHE includes only one acidic residue, while SHED includes both (Fig. 5b, e). As depicted in Fig. 5c, f, the barriers of SHD are higher than that of SHE and SHED both in acylation and deacylation, while the barriers of SHED are the lowest (see Table 1) and quite close to each other for each step. Deacylation remains in all cases the rate-limiting step, as suggested experimentally by the similar *k*_cat_ of WT with different substrates^10^. Interestingly, during acylation (Supplementary Fig. 3) the H408 Nδ1-H temporarily transfers to D356 Oδ1, aiding proton abstraction from S267.

**Table 1:** Δ*G* (kcal/mol) against initial proton transfers in acylation and deacylation of WT *Ot*CE15A and *Ot*CE15A–D356A, –E290A, and –R268A variants with the MeGlcA substrate using units of SHE, SHD, and SHED, respectively.

	WT-SHE	WT-SHD	WT-SHED	D356A-SHE	E290A-SHD	R268A-SHED
Acylation	10.0 ± 1.1	19.1 ± 1.7	6.8 ± 0.6	9.3 ± 0.3	19.2 ± 1.7	9.6 ± 0.8
Deacylation	14.4 ± 0.3	29.8 ± 1.5	7.5 ± 0.7	18.4 ± 0.4	32.0 ± 0.7	>110

The given error is the standard error inferred from three independent runs (see Figs. 2, 4 and 5). Source data are provided as a Source Data file (see individual figures)^*^.

^*^QM area of WT/D356A-SHE and WT/E290A-SHD includes one acidic residue, i.e., Glu290 and Asp356, respectively (total charge: −1), while SHED consists of two acidic residues—Glu290 and Asp356 (total charge: −2). The QM atoms for WT/D356A-SHE, WT/E290A-SHD, and SHED are 48, 48, and 54 in acylation, and 45, 45, and 51 in deacylation, respectively.

We also attempted to introduce the R268 side chain, the proposed oxyanion hole residue, in the QM region. In this simulation, we found that the proton in Arg268 transferred to the carbonyl group of the substrate (Supplementary Fig. 4a). However, the expected back-transfer of this proton does not occur during simulation time, obstructing further progress of the reaction, with sharp free-energy increase (Supplementary Fig. 4b). We conclude that a reaction itinerary involving at least five potentially key players such as in the SHEDR assembly cannot be adequately handled with the given methodology. Full description of complex, multidimensional reaction itineraries, without presumption of reaction coordinates, would require prohibitively costly computational strategies. Thus, we continued our investigations with the SHED assembly as best realistic choice.

### Determination of the experimental *E*_a_ by Arrhenius analysis

**Fig. 6:**
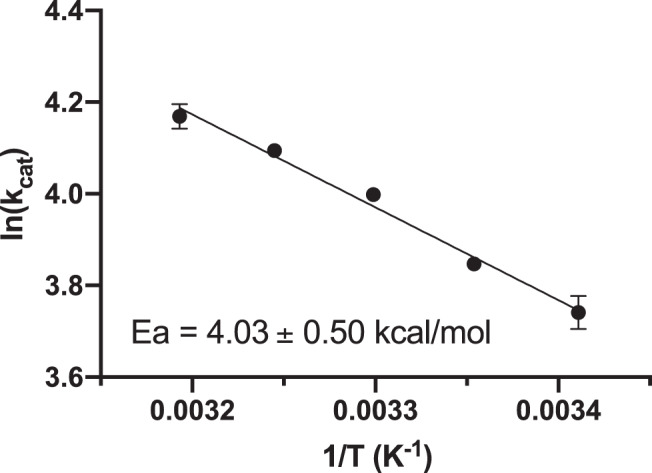
Arrhenius activation energy determination of the *Ot*CE15A BnzGlcA cleavage reaction. Assays for the determination of Michaelis constants were carried out in duplicate at 5 °C intervals from 20 to 40 °C from a single batch of purified enzyme as described in the “Methods” section. Errors represent the standard error of the mean for *k*_cat_ values determined by non-linear regression.

In order to obtain an additional term of comparison for our calculations, we determined the experimental activation energy of the overall reaction with the small substrate benzyl GlcA (BnzGlcA) by determining *k*_cat_ at different temperatures. *E*_a_ was determined as 4.03 ± 0.5 kcal/mol (Fig. 6). Considering the differences in the experimental and computational set ups, we find it encouraging that this is within 2-fold of the calculated value of 7.5 ± 0.7 kcal/mol barrier for deacetylation of MeGlcA by the WT-SHED assembly, also highlighting that this is the most appropriate assembly among the tested ones.

### Investigating the role of the acidic residues by in silico mutation

In our simulations, the residues in the MM area can interact electrostatically with the ones in the QM area. The acidic residues can accordingly play roles in catalysis by electrostatic interactions when they were included in MM area. Two assemblies of *Ot*CE15A were constructed to further probe the roles of the acidic residues using catalytic units SHE and SHD with D356A and E290A variants, respectively. As shown in Table 1 and Supplementary Fig. 5, the barriers of D356A-SHE and E290A-SHD against the first proton transfer in acylation are similar to that of the corresponding wild types (WTs). However, the barriers of the variants are 4 and 2.2 kcal/mol higher than that of their WTs in deacylation, quantifying the role of the acidic residues in stabilizing the reaction electrostatically. These results are qualitatively in line with our previously published results showing decreases in *k*_cat_ both with E290A and D356A substitutions using BnzGlcA as substrate^10^. However, the experimentally measured effects for single substitutions were at most a ~3-fold decrease in *k*_cat_ for D356A (see reference values in Table 2) while a more substantial decrease would be expected from the calculations. Experimental substitution of both residues does cause a much larger decrease of in *k*_cat_ (~85-fold). The discrepancies between experiment and calculations suggest that additional compensatory mechanisms in the enzyme are not taken into account by the calculations, such as changes in the surrounding residues’ p*K*_a_ caused by removal of negative charges.

**Table 2:** Kinetic parameters of *Ot*CE15A variants from this (marked with *) and previous work^9,10^.

Enzyme	*K*_m_ (mM)	*k*_cat_ (s^−1^)	*k*_cat_/*K*_m_ (s^−1^ M^−1^)
*BnzGlcA*			
Wild Type	3.57 ± 0.091	16.6 ± 0.110	4.65E+03 ± 1.2E+02
S267A	2.83 ± 0.660	0.000983 ± 0.000059	3.47E−01 ± 8.3E−02
H408A	0.184 ± 0.035	0.00999 ± 0.000400	5.43E+01 ± 1.1E+01
E290A	2.03 ± 0.110	10.3 ± 0.110	5.07E+03 ± 2.8E+02
D356A	1.86 ± 0.110	5.21 ± 0.073	2.80E+03 ± 1.7E+02
E290A/D356A	0.502 ± 0.034	0.196 ± 0.003	3.90E+02 ± 2.7E+01
R268A*	0.408 ± 0.051	0.204 ± 0.007	5.01E+02 ± 6.5E+01
*MeGlcA*			
Wild Type*	2.77 ± 0.150	19.0 ± 0.310	6.85E+03 ± 3.9E+02
R268A*	0.386 ± 0.060	0.214 ± 0.009	5.54E+02 ± 9.3E+01

The error on the kinetic parameters was calculated by non-linear regression based on duplicate measurements at 8 substrate concentrations.

### Proposed role of conserved active-site arginine as oxyanion hole

**Fig. 7:**
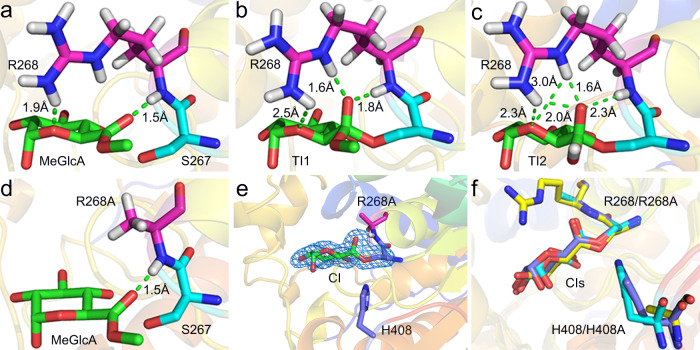
Role of active-site Arg268. **a** Starting structure of *Ot*CE15A (PDB 6SYR) with MeGlcA. **b**, **c** Snapshots of TI1 and TI2 in our simulations with Arg268. **d** Starting structure of *Ot*CE15A-R268A variant in silico. **e** Difference omit electron density of CI in crystal structure of *Ot*CE15A-R268A (PDB 7B7H) generated experimentally here (contoured at 4σ level). **f** Comparison of CI in *Ot*CE15A-R268A generated from our simulation (cyan) with the two experimental ones, i.e., *Ot*CE15A-R268A (blue) and *Ot*CE15A-H408A (PDB 6SZ4, yellow)^9^ variants. In **a**–**e**, C atoms of Arg268 and R268A are highlighted in pink. C atoms of MeGlcA are shown in green. C atoms of catalytic residues generated from simulation and from crystal structure are shown in cyan and blue, respectively. In **f**, the C atoms in the structure of the R268A simulation, and crystal structures of the two variants—R268A and H408A -  are colored in cyan, blue and yellow, respectively.

In many ABHs, the oxyanion hole is solely contributed by main chain atoms^47^, including the main chain amide of the residue immediately following the nucleophile. In contrast, in GEs the side chain (in addition to main chain) of the conserved Arg (Arg268 in *Ot*CE15A) following the nucleophilic Ser, is thought to contribute significantly to stabilization of the transition state^10^. There is at least one additional example of an ABH where an Arg side chain has been implicated in the oxyanion hole stabilization^48^, but in this case the Arg is contributed from a helix facing the active site. Indeed, during the simulations with the SHED catalytic assembly, compared with the starting point structure (Fig. 7a), more hydrogen bonds were found to be formed between Arg268 and the carboxylate oxygen in transient states of acylation (Fig. 7b) and deacylation (Fig. 7c), especially the latter. As described above, we did not obtain fully meaningful simulation results in the given time scale using SHEDR unit as the QM area. To further probe the role of R268, the R268A variant was constructed in silico (Fig. 7d) in the SHED catalytic assembly as the QM region, which can in our simulations interact electrostatically with the MM area. The barrier against initial proton transfer accompanied by the nucleophilic attack in acylation of *Ot*CE15A-R268A with catalytic unit SHED was 9.6 kcal/mol (see Table 1 and Supplementary Fig. 6).

However, in the deacylation step of the R268A variant, the proton transfer was not accompanied by nucleophilic attack. Furthermore, in the specified time scale, the proton cannot complete its transfer process to His408, as the corresponding barrier is exceedingly large (see Table 1). This suggests that the CI would easily accumulate in this variant. The *Ot*CE15A-R268A variant was constructed experimentally and kinetic parameters determined with both BnzGlcA and MeGlcA (Table 2). With both substrates the *k*_cat_ is reduced >80-fold compared to the WT enzyme, and there is also reduction in *K*_m_ as observed previously with the H408A variant, which would be consistent with accumulation of the CI. Indeed, crystallographic determination of the *Ot*CE15A-R268A structure (Supplementary Table 1) after soaking with the BnzGlcA substrate clearly shows the formation of a CI (Fig. 7e), mainly arising from the significantly damaged interactions in TI2 (Fig. 7c). Thus, the experimental results are in qualitative agreement with the calculations. The CI in the crystal structure of *Ot*CE15A-R268A was compared with the one generated from our simulation and the one of *Ot*CE15A-H408A variant (Fig. 7f), showing little differences.

### Dissociation action

On a natural substrate, after a lignin fragment has been expelled from the active site, GEs will undergo a dissociation process of the EP (enzyme-product) complex to complete the hydrolysis cycle. This process, unlike the catalytic event itself, is very likely affected by the properties of the saccharide portion of the substrate. In some biomass degrading enzymes, the presence of carbohydrate-binding modules (CBMs) will also affect this process, but *Ot*CE15A is naturally devoid of a CBM. In the construct used for both structural and biochemical characterization, only 15 N-terminal residues from the original sequence were removed, and these were predicted to be unstructured and shown experimentally not to alter the enzyme kinetics on model substrates.

**Fig. 8:**
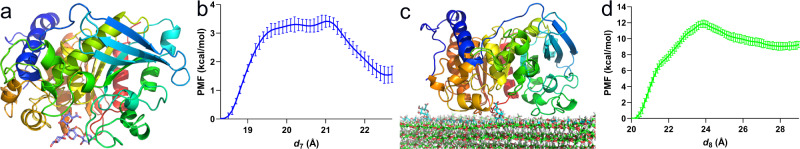
Enzymatic dissociation processes from model and complex substrates. **a**, **c** GE with **a** XUX (PDB 6T0I) and **c** NSC. **b**, **d** Corresponding free-energy profiles characterizing the dissociation processes. C atoms of XUX are highlighted in blue. In NSC, cellulose and xylan with GlcA are colored in green and cyan, respectively. *d*_7_ and *d*_8_ denote the distance of COMs between enzyme and substrate. Data are presented as mean values ± the standard error inferred from three independent runs. Source data for **b**, **d** are provided as a Source Data file.

As depicted in Fig. 8a, we previously obtained a complex of *Ot*CE15A with the largest model ligand to date in a CE15 protein structure—the tetrasaccharide 2^3^-(4-*O*-methyl-α-d-glucuronyl)-xylotriose (XUX)^10^. This crystal complex was used as the starting structure to investigate the process of dissociation between enzyme and model ligand. In the present work, a natural substrate complex (NSC), consisting of an 18-chain Iβ cellulose fibril with a 2-3-4-4-3-2 habit^49^ and a hemicellulose chain adsorbed at the (110) face was constructed in silico. Six cellulose chains at the bottom of the fibril were removed for simplicity. The adsorbed xylan chain is decorated with α-(1,2)-MeGlcA branches every sixth xylosyl unit, and the entire chain adopts a 2-fold helical screw conformation, based on the findings of Dupree and co-workers (Fig. 8c)^10,50–52^. Referring to the binding model between *Ot*CE15A and XUX in the crystal structure, the complex of enzyme with NSC was constructed and used as the starting model for the following calculation of dissociation. Fifteen residues in *Ot*CE15A, found in the interaction interface and including the active site residues, interact with NSC by hydrogen bonds (Supplementary Fig. 7). The processes of dissociation between enzyme and substrates were then investigated in silico.

As shown in Fig. 8b, d, the barriers for assemblies of XUX and NSC are approximately 3.3 and 12 kcal/mol, respectively, indicating that the enzymatic dissociation rate from NSC will be much slower than that from the smaller and soluble XUX molecule. We suggest that, depending on the exact LCC structure being attacked, different steps of the catalytic cycle can be rate limiting for GEs, including dissociation of the EP complex. Thus, the rate-limiting step needs to be carefully considered in protein engineering programs on GEs, and generally in the analysis of variants. In particular, improvement of activity on smaller model substrates commonly used, may not necessarily result in improvement on real biomass, where the nature of the LG or non-catalytic processes of dissociation (and perhaps association) may alter the rate-limiting step.

## Discussion

Based on our previously obtained considerable biochemical and structural information, in the present work we shed light on the catalytic mechanism and biomass association of *Ot*CE15A, which has a distinctive variation on the classical serine hydrolase catalytic triad. The roles of the two putative acidic residues which were shown to be important for catalysis experimentally^10^, were quantified in silico by using different QM regions and relevant Ala mutations as MM areas. Furthermore, our theoretical results indicated that the conserved active-site arginine, whose side chain is proposed to contribute to forming the oxyanion hole, plays a prominent role in both acylation and deacylation steps, especially in the latter. This is reflected in a large reduction in activity of the R268A mutant as produced experimentally here and accumulation of the CI in the variant demonstrated in the determined X-ray structure. The clear roles of these key residues in catalysis expand and deepen our knowledge on the classical catalytic triad of serine hydrolases, and can contribute to the improvement of designed biomimetics based on it. In addition, the QM/MM calculations based on the assumption of a classic ABH mechanism, confirm deacylation to be the rate-determining step in all the six different assemblies, with the initial proton transfer being the energetically dominant process for both the two catalytic steps. The lowest and probably most reliable energy barriers for the WT *Ot*CE15A are obtained with a QM region including catalytic Ser, His and two acidic residues. The barrier of the deacylation step as calculated for this WT-SHED assembly is also the closest to our experimentally determined *E*_a_. Finally, the calculations suggest that with bigger substrates, non-catalytic events, such as dissociation between the enzyme and its final product can become rate-limiting, and this needs to be carefully considered in engineering GEs for biomass applications. This work, at the confluence of computation and experiment, is expected to help drive the use of GEs as important and perhaps decisive accessory enzymes to help reduce the inherently high recalcitrance of lignocellulose in a more cost-effective and environment friendly way.

## Methods

### QM/MM free-energy calculations

The PMFs of acylation, deacylation and angle change of the HW were determined for *Ot*CE15A in aqueous solution by QM/MM simulations. The starting structure for the simulations is the complex of *Ot*CE15A with MeGlcA. The QM region for acylation of WT-SHE, which has an overall charge of −1, includes Ser267, Glu290, His408, as well as the MeGlcA ligand (48 atoms). To investigate the roles of acidic residues and R268 in acylation, catalytic assemblies of WT-SHD, WT-SHED, WT-SHEDR, D356A-SHE and E290A-SHD and R268A-SHED were used. Their QM areas consist of 48 (charge: −1), 54 (charge: −2), 66 (charge: −1), 48 (charge: −1), 48 (charge: −1) and 54 (charge: −2) atoms, respectively. The QM areas of WT-SHE for deacylation and the angle change of HW, which have the overall charge of −1, contain the atoms of WT-SHE for acylation (except for the LG) and a HW (45 atoms). Catalytic assemblies of WT-SHD, WT-SHED, WT-SHEDR, D356A-SHE and E290A-SHD and R268A-SHED in deacylation consist of 45 (charge: −1), 51 (charge: −2), 63 (charge: −1), 45 (charge: −1), 45 (charge: −1) and 51 atoms (charge: −2), respectively.

All the assemblies were solvated in an equilibrated box of water, and the overall charge neutrality was achieved by adding Na^+^ ions to the solution. The total molecular numbers for each assembly were about 63,250 atoms. Each complex was optimized by 5000 steps minimization and 100 ps molecular dynamics (MD) simulation with gradually relaxing restraints: (a) protein and substrate were restrained; (b) only substrate and catalytic residues were restrained; and (c) 1000 steps minimization was performed before each run without restraint. Then three independent runs of the QM/MM simulation for each molecular assembly were carried out. Furthermore, the transition pathways were stratified for increasing the efficiency of the calculations (see Supplementary Table 2). A recently developed importance-sampling algorithm, WTM-eABF^53–56^, implemented in the Colvars^57^ module of the NAMD^58^ molecular dynamics program, was employed in all the free-energy calculations. MOPAC35^59^ and NAMD 2.13 were used as the QM and MD engines, respectively. PM7^60^ was utilized in the QM calculations, wherein electrical embedding was used to treat Coulombic interactions at the QM/MM interface^61,62^. The PM7 semi-empirical method provides a good balance between accuracy and efficiency in QM/MM calculations. Apart from benchmarking^63^, the reliability of PM7 for modeling the catalytic mechanism in enzymes has been assessed previously^64^. In a previous study, we have successfully combined PM7 with the WTM-eABF method to study the proton transfer in the glycosylation of the catalytic cycle of glycoside hydrolase^46^. Therefore, PM7 is suitable for systems that are not particularly complex. All the simulations for these assemblies were carried out using NAMD with the CHARMM36 force field^65–67^, and the TIP3P water model^68^. VMD 1.9.4 program^69^ and PyMOL molecular graphics system (version 2.4 Schrödinger, LLC) were employed to visualize and analyze results.

### Free-energy calculations

The assemblies for the investigation of the methanol group leaving the active site, HW approach and dissociation action from XUX and NSC were treated as described above. The free-energy profiles underlying these processes were calculated via WTM-eABF. The details for each molecular assembly are provided in Supplementary Table 3. All the free-energy profiles reported in this study were obtained over three independent WTM-eABF simulations.

### Enzyme production and characterization

The *Ot*CE15A R268A variant was created by site-specific mutagenesis via QuikChange by the primers listed in Supplementary Table 4. The variant was recombinantly produced in *E. coli* BL21 (λDE3) and purified by immobilized metal affinity chromatography and subsequently by size-exclusion chromatography as described previously^9,10^. Glucuronoyl esterase activity was assayed with BnzGlcA or MeGlcA (Carbosynth) and monitored continuously with the K-URONIC kit (Megazyme) as reported previously^9,10^. Initial velocity measurements at defined substrate concentrations were carried out in duplicate from a single batch of recombinantly purified enzyme and kinetic parameters were determined from at least eight different substrate concentrations ranging from 0.1 to 10 times the *K*_m_ value. Determination of the Arrhenius activation energy (*E*_a_) for the BnzGlcA cleavage reaction was carried out similarly as described above at temperatures ranging from 20 to 40 °C. Reaction conditions containing all the components, except substrate, were pre-equilibrated at the defined temperature for 5 min before starting the assays. Substrate was kept at room temperature and added in volumes ≤5% of the total volume to ensure minimal temperature disruption. Reactions were monitored continuously in a SPECTRO star nano spectrophotometer (BMG Labtech) equipped with controlled thermal incubation^9,10^.

### Crystal structure determination

The *Ot*CE15A R268A variant was crystallized using a Morpheus screen (Molecular Dimensions Limited, Sheffield, UK). Sitting drops of 0.3 μL total volume were set up in MRC two-drop crystallization plates (Molecular Dimensions) with protein at concentration of 7.1 mg/mL in a standard chromatography buffer (50 mM Tris–HCl pH 8.0, 100 mM NaCl) mixed with reservoir solutions in either a 3:1 or 1:1 ratio with an Oryx8 robot (Douglas Instruments, East Garston, UK). Crystals used for data collection were obtained in condition 2-38 of the Morpheus screen (https://www.moleculardimensions.com/products/morpheusv3) consisting of 0.1 M buffer system 1 (imidazole and MES, pH 6.5), 30% precipitant mix 2 (ethylene glycol and PEG 8000) and amino acid additives (l-glutamate, alanine (racemic); glycine; lysine HCl (racemic); serine (racemic)). Soaking was carried out in a saturated solution of BnzGlcA in 10% DMSO and 90% mother liquor for 30 s before being flash frozen in liquid N_2_. Diffraction data was collected at the BioMAX^70^ beamline at MAX IV (February 23, 2020). The dataset was processed with XDS (version 20200131)^71^ and the structure solved by molecular replacement with Phaser 2.1^72^ using the WT *Ot*CE15A (PDB code 6GS0) as a template. Molecular replacement was utilized as rigid body refinement in Phenix Refine^73^ failed to correctly determine phases which was likely attributable to differences in the cell dimensions between the WT and *Ot*CE15A-R268A dataset. Coot 0.8.9.2^74^ and Phenix Refine^73^ (Phenix 1.19.2-4158-000) were used in iterative cycles of real space and reciprocal space refinement. Two stretches of residues (154–175 and 385–388) and the residues from the protein expression His_6_-tag were not modeled due to a lack of electron density likely owing to soaking in the presence of DMSO as has been observed in crystal soaks in our previous study^10^. The data collection, processing, and refinement statistics can be found in Supplementary Table 1. Data was collected on BioMAX beamline at the MAXIV facility in Sweden on February 23, 2020.

### Reporting summary

Further information on research design is available in the Nature Research Reporting Summary linked to this article.

## Supplementary information

Supplementary Information

Description of Additional Supplementary Files

Supplementary Movie 1

Supplementary Movie 2

Supplementary Movie 3

Supplementary Movie 4

Reporting Summary

## Data Availability

The data that support this study are available from the corresponding author upon reasonable request. Crystallographic data for the *Ot*CE15A R268A variant have been deposited at the Protein Data Bank with accession code 7B7H. Previously released structural data used in the course of this study: 6SYR, 6SZ4, 6GS0, and 6T0I. Source data are provided with this paper.

## Source data

Source Data
