# Supplementary material for: Mechanism and biomass association of glucuronoyl esterase: an α/β hydrolase with potential in biomass conversion
Source: Nat Commun. 2022 Mar 18;13:1449. doi: 10.1038/s41467-022-28938-w (PMC8933493; doi:10.1038/s41467-022-28938-w)
Supplement: Supplementary file 1 — Supplementary Information [file 41467_2022_28938_MOESM1_ESM.pdf]

Supplementary information for

Mechanism and biomass association of Glucuronoyl Esterase, an α/β hydrolase with potential in biomass conversion

Zhiyou Zong [1, 2], Scott Mazurkewich [3], Caroline S. Pereira [4], Haohao Fu [2], Wensheng Cai [2], Xueguang Shao [2], Munir S. Skaf [4], Johan Larsbrink [3] and Leila Lo Leggio [*, 1]

[1] *Department of Chemistry, University of Copenhagen, DK-2100 Copenhagen, Denmark.*

[2] *College of Chemistry, Research Center for Analytical Sciences, Tianjin Key Laboratory of Biosensing and Molecular Recognition, State Key Laboratory of Medicinal Chemical Biology, Nankai University, Tianjin 300071, P. R. China.*

[3] *Wallenberg Wood Science Center, Division of Industrial Biotechnology, Department of Biology and Biological Engineering, Chalmers University of Technology, SE-412 96 Gothenburg, Sweden.*

[4] *Institute of Chemistry and Center for Computing in Engineering and Sciences, University of Campinas – Unicamp, Campinas, SP, 13084-862, Brazil.*

***Corresponding E-mail:** *leila@chem.ku.dk*

Supplementary Table 1 Crystallographic statistics for the R268A variant of *Ot*CE15A in complex with GlcA. Data in parenthesis is for the highest resolution shell.

Data Collection	
Wavelength (Å)	1.008
Space group	P1
Cell dimensions	
a, b, c (Å)	47.33, 47.30, 51.11
α, β, γ (°)	61.95, 67.92, 88.27
No. of measured reflections	114935 (11273)
No. of independent reflections	32171 (3093)
Resolution (Å)	43.15 - 1.80 (1.86 - 1.80)
R_{merge}[1]	0.12 (1.25)
$CC_{1/2}$ (%)	99.5 (46.1)
Mean I/σI	6.84 (0.95)
Wilson B-factor	25.74
Completeness (%)	96.6 (93.0)
Redundancy	3.6 (3.6)
Refinement	
R_{work}/R_{free}	0.182/0.222
No. atoms	
Protein	2906
Ligand/ions	106
Water	111
Average B-factors	
Protein	31.9
Ligand/ions	45.8
Water	37.8
Ramachandran statistics[2]	
Favored (%)	97.8
Allowed (%)	2.2
Outliers (%)	0.0
RMSD from ideal geometry[3]	
Bond length (Å)	0.007
Bond angles (°)	0.90
PDB accession	7B7H

[1] $R_{merge} = \sum_{hkl}\sum_i |I_i(hkl) - \langle I(hkl)\rangle| / \sum_{hkl}\sum_i I_i(hkl)$, wherein $I_i(hkl)$ is the intensity of the *i*th measurement of reflection *hkl*, and $\langle I(hkl)\rangle$ is the mean value of $I_i(hkl)$ for all the *i* measurements.

[2] Calculated by Phenix Refine[1].

[3] Root mean square deviations from ideal geometry values[2].

Supplementary Table 2 Details of the molecular assemblies for the calculations of the PMFs describing the catalytic reaction by QM/MM simulations.*

No.	Molecular assemblies	Number of atoms	Size of the simulation box (Å^3)[a]	Processes*	Simulation time (ps)	Runs[b]
**1**	6SYR[c]	63,254	85×86×92	IPTA	18	3
**2**	6SYR[d]	63,254	85×86×92	FPTA	30	3
**3**	6SYR[e]	63,254	85×86×92	ACHW	12	3
**4**	6SYR[f]	63,254	85×86×92	IPTD	24	3
**5**	6SYR[g]	63,254	85×86×92	FPTD	30	3
**6**	6SYR[h]	63,254	85×86×92	IPTA-SHD	18	3
**7**	6SYR[i]	63,254	85×86×92	IPTA-SHED	48	3
**8**	6SYR[j]	63,254	85×86×92	IPTD-SHD	24	3
**9**	6SYR[k]	63,254	85×86×92	IPTD-SHED	60	3
**10**	6SYR-D356A[l]	63,254	85×86×92	D356A-IPTA-SHE	18	3
**11**	6SYR-D356A[l]	63,254	85×86×92	D356A-IPTD-SHE	24	3
**12**	6SYR-E290A[m]	63,251	85×86×92	E290A-IPTA-SHD	18	3
**13**	6SYR-E290A[m]	63,251	85×86×92	E290A-IPTD-SHD	24	3
**14**	6SYR- R268A[n]	63,244	85×86×92	R268A-IPTA-SHED	18	3
**15**	6SYR- R268A[n]	63,244	85×86×92	R268A-IPTD-SHED	24	3
**16**	6SYR[o]	63,254	85×86×92	IPTA-SHEDR	48	2
**17**	6SYR[o]	63,254	85×86×92	IPTD-SHEDR	60	2

* The simulations were carried out with *cutoff* of 12.0 Å, *fullsamples* of 10, *hillweight* of 0.1 kcal/mol, *hillwidth* of 3 Å and *biastemperature* of 3000 K. A small time step of 0.02 fs was used for each assembly to capture the fast bond-breaking and forming processes. The WTM-eABF method can enhance the sampling of the free energy landscape, facilitating reaction to complete in the corresponding timescales. Furthermore, three parallel simulations show error bars with a maximal value of 1.7 kcal/mol, indicating that our free-energy calculations have been converged.

[a] The size of the solvent boxes guarantees a minimum distance of 15 Å from any atom of the enzyme to any edge of the simulation box.

[b] Independent QM/MM runs.

[c] Initial structure of *Ot*CE15A and methyl-glucuronate (MeGlcA) built by adding a methyl group to the glucuronate complex (PDB code 6SYR) for investigation of the initial proton transfer in acylation (IPTA). The QM region consists of 48 atoms, including Ser267, Glu290, His408, as well as the MeGlcA.

[d] The structure generated from the trajectory of assembly 1 in Supplementary Table 2 is used as the initial model for investigation of final proton transfer in acylation (FPTA).

[e] The structure corresponding to the minimum of the PMF, generated from the trajectory of assembly 2 in Supplementary Table 3, is used as the initial model for angle change of hydrolytic water (ACHW).

[f] The structure generated from the trajectory of assembly 3 in Supplementary Table 2 is used as the initial model for investigation of the initial proton transfer in deacylation (IPTD). The QM region consists of 45 atoms, containing the QM atoms of the assembly 1 (except for the leaving group, i.e., LG) and a hydrolytic water.

[g] The structure generated from the trajectory of assembly 4 in Supplementary Table 2 is used as the initial model for investigation of final proton transfer in deacylation (FPTD).

[h] Initial structure of assembly 1 in Supplementary Table 2 for investigation of IPTA with one acidic residue–Asp356 (unit SHD). The QM region consists of 48 atoms (total charge: -1), including Ser267, Asp356, His408, as well as MeGlcA.

[i] Initial structure of assembly 1 in Supplementary Table 2 for investigation of IPTA with two acidic residues–Glu290 and Asp356 (unit SHED). The QM region consists of 54 atoms (total charge: -2), including Ser267, Glu290, Asp356, His408, as well as MeGlcA.

[j] Initial structure of assembly 6 in Supplementary Table 2 for investigation of IPTD with one acidic residue–Asp356 (unit SHD). The QM region consists of 45 atoms (total charge: -1).

[k] Initial structure of assembly 7 in Supplementary Table 2 for investigation of IPTD with two acidic residues–Glu290 and Asp356 (unit SHED). The QM region consists of 51 atoms (total charge: -2).

[l] Initial structure of assembly 1 in Supplementary Table 2 with D356A variant for investigation of the IPTA and IPTD with catalytic unit SHE.

[m] Initial structure of assembly 1 in Supplementary Table 2 with E290A variant for investigation of the IPTA and IPTD with catalytic unit SHD.

[n] Initial structure of assembly 1 in Supplementary Table 2 with R268A variant for investigation of the IPTA and IPTD with catalytic unit SHED.

[o] Initial structure of assembly 1 in Supplementary Table 2 for investigation of IPTA and IPTD using SHED(R) unit. The QM region consists of 66 and 63 atoms respectively.

Supplementary Table 3 Details of the molecular assemblies for free-energy calculations in this study.*

No.	Molecular assemblies	Number of atoms	Size of the simulation box (Å^3)[a]	Processes*	Simulation time (ns)	Runs[b]
**1**	6SYR[c]	63,254	85×86×92	MGL	100	3
**2**	6SYR[d]	63,254	85×86×92	HWA	100	3
**3**	6T0I[e]	60,418	82×86×92	ED-XUX	200	3
**4**	6T0I[e]	80,325	86×85×119	ED-NSC	360	3

* Assembly 1 for investigation of the methanol group leaving (MGL) the active site; assembly 2 for investigation of hydrolytic water approach (HWA) the active site. Assembly 3 and 4 for investigation of the enzymatic dissociation (ED) from substrates. The time steps for the simulations are 2.0 fs.

[a] The size of the solvent boxes guarantees a minimum distance of 15 Å from any atom of the enzyme to any edge of the simulation box.

[b] Independent MD runs.

[c] The structure generated from the trajectory of assembly 2 in Supplementary Table 2 is used as the initial model for investigation of MGL process.

[d] The structure generated from the trajectory of assembly 1 in Supplementary Table 3 is used as the initial model for investigation of the HWA process.

[e] Initial structure of *Ot*CE15A (PDB code 6T0I) with XUX and natural substrate complex (NSC) for investigation of ED processes, namely ED-XUX and ED-NSC, respectively. The NSC underwent 5000 step minimization and 100 ps MD simulation before construction of *Ot*CE15A-NSC complex. The complex was pretreated with 5000 steps minimization and 100 ps MD simulation without restraint, and further with 100 ns equilibrium with restraint of heavy atoms in NSC.

Supplementary Table 4 Primers utilized for creation of *Ot*CE15A-R268A variant.

Gene	Primer<br>5'-3' sequence
*Ot*CE15A-R268A	F: GCATTCGGCGCTCGGCAAG
	R: GCCGAGCGCCGAATGCC

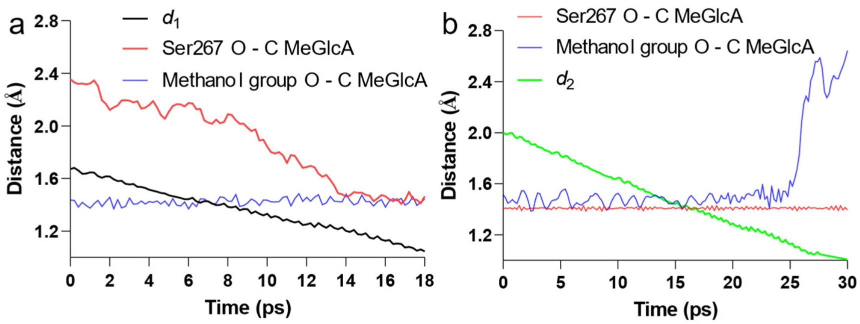

Supplementary Fig. 1 Distances analysis. a and **b** Monitor of the forming of TI1, covalent intermediate and leaving group. d_1 denotes the distance between the proton in Ser267 and Nε of His408. Ser267 O - C MeGlcA denotes the distance between the nucleophilic O in Ser267 and attacked carbonyl C of MeGlcA which is nucleophilic attack, showing progressive shortening with formation of the covalent bond. Methanol group O - C MeGlcA denotes the distance between the leaving methanol group O and the carbonyl C in MeGlcA in the cleaved ester bond, showing sudden lengthening on leaving of the group. d_2 denotes the distance between the proton of Nε His408 and O in methanol group of MeGlcA.

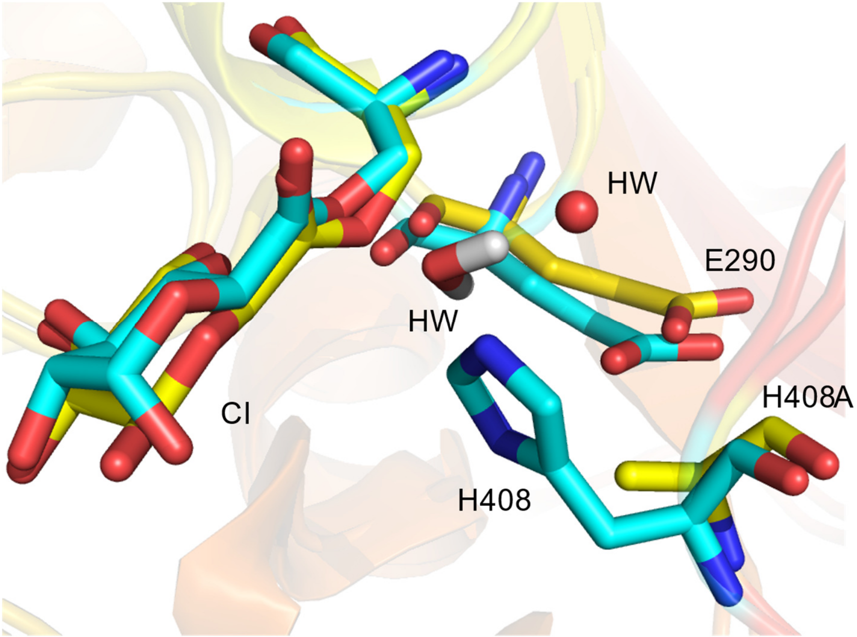

Supplementary Fig. 2 The comparison of CI in H408A variant (PDB code 6SZ4) with the one generated from our simulation. The C atoms of the former and the latter are colored in yellow and cyan, respectively. Water molecules near the active site are shown in sphere (Wat 704 in 6SZ4) and stick (simulation), respectively. Our computational CI aligns well with the experimental one. In the experimental complex we observe a β-anomer, however we choose to use the α-anomer in simulation to better reflect the α-linkage of the glucuronic acid to xylan in the natural substrate.

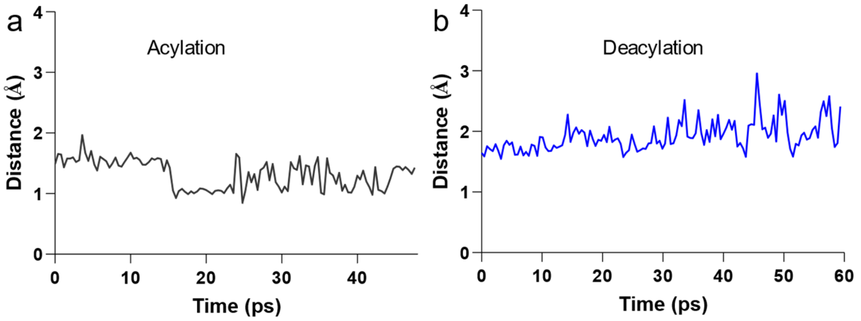

Supplementary Fig. 3 Distance monitoring between H408 Nδ1-H and D356 Oδ1 in (a) acylation and (b) deacylation steps in SHED unit as the QM area.

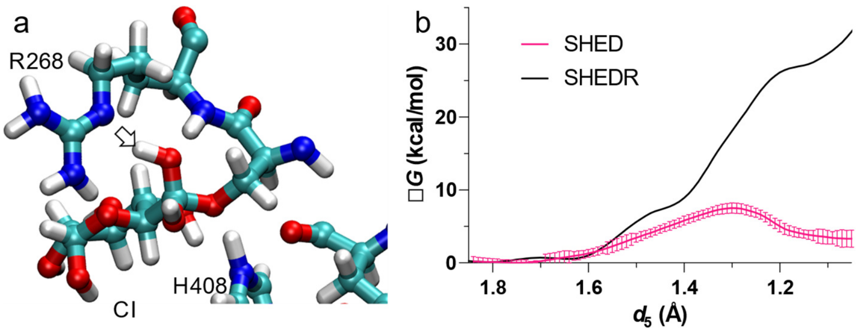

Supplementary Fig. 4 QM/MM simulations for SHEDR unit as the QM area. (a) Structure obtained from the trajectory of the QM/MM simulation in deacylation step, and (b) the relevant free-energy profiles, wherein the one of SHED unit (pink line) is used as a comparison. This calculation was repeated twice, with the average value given in b.

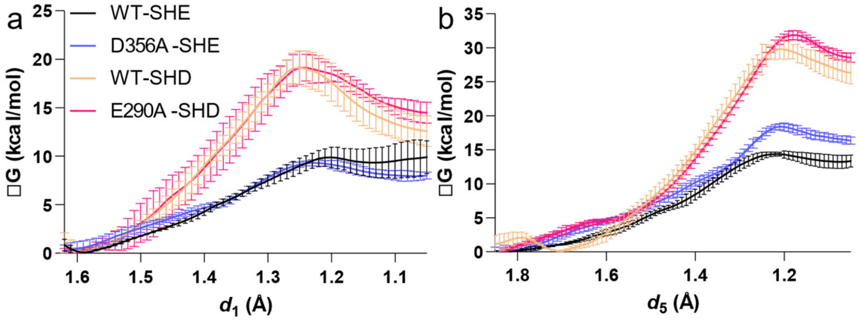

Supplementary Fig. 5 Free-energy profiles generated from QM/MM simulations for assemblies of D356A-SHE and E290A-SHD in the first proton transfer processes. a Acylation. **b** Deacylation. The results of WT-SHE and WT-SHD are shown for reference. Data are presented as mean values +/- the standard error inferred from three independent runs. Source data are provided as a Source Data file.

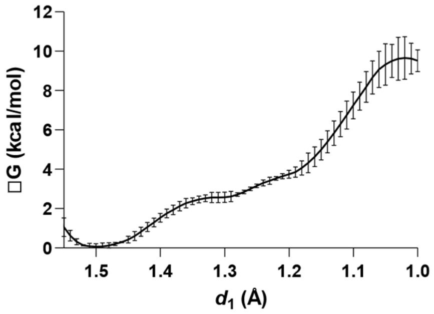

Supplementary Fig. 6 The PMF calculated along the coordinate of d_1 for assembly of *Ot*CE15A-R268A with catalytic unit SHED. Owing to the damaged electrostatic interactions in TI1 by R268A, the reaction becomes unstable, resulting in higher barrier than that of WT. Data are presented as mean values +/- the standard error inferred from three independent runs. Source data are provided as a Source Data file.

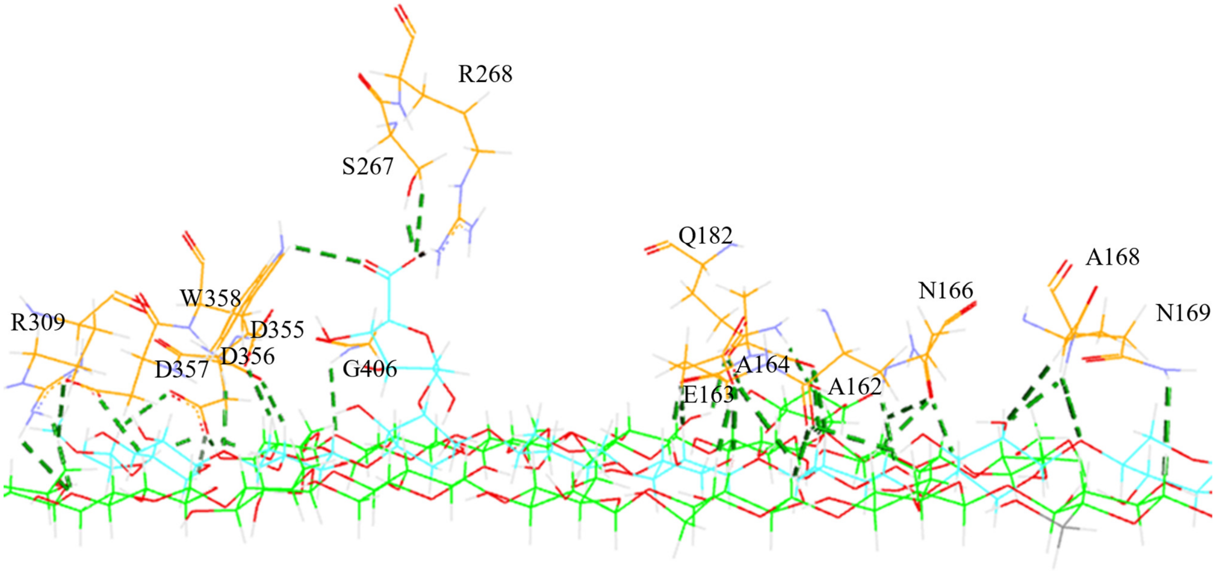

Supplementary Fig. 7 Hydrogen bonding (green dotted line) interactions of the residues in *Ot*CE15A (Ala162, Glu163, Ala164, Asn166, Ala168, Asn169, Gln182, Ser267, Arg268, Arg309, Asp355, Asp356, Asp357, Trp358, Gly406) with the natural substrate. C atoms of residues in *Ot*CE15A are colored orange, while C atoms of cellulose and glucuronoxylan (xylose and GlcA) in natural substrate are in green and cyan, respectively.

References

(1) Liebschner, D., Afonine, P. V., Baker, M. L., Bunkóczi, G., Chen, V. B., Croll, T. I., Hintze, B., Hung, L.W., Jain, S. & McCoy, A. J. et al. Macromolecular structure determination using X-rays, neutrons and electrons: recent developments in phenix. *Acta Cryst.* **75**, 861−877 (2019).

(2) Engh, R. A., & Huber, R. Accurate bond and angle parameters for X-ray protein structure refinement. *Acta Crystallographica Section A* **47**, 4 (1991).
