# Supplementary material for: Mechanism and biomass association of glucuronoyl esterase: an α/β hydrolase with potential in biomass conversion
Source: Nat Commun. 2022 Mar 18;13:1449. doi: 10.1038/s41467-022-28938-w (PMC8933493; doi:10.1038/s41467-022-28938-w)
Supplement: Supplementary file 2 — Description of Additional Supplementary Files [file 41467_2022_28938_MOESM2_ESM.pdf]

Description of Additional Supplementary Files

File Name: Supplementary Movie 1
Description: Initial proton transfer from S267 to Nε His408 and relevant nucleophilic attack in acylation.

File Name: Supplementary Movie 2
Description: Proton transfer from Nε His408 to create CI and leaving group in acylation.

File Name: Supplementary Movie 3
Description: Initial proton transfer from hydrolytic water and relevant nucleophilic attack on CI in deacylation.

File Name: Supplementary Movie 4
Description: Proton transfer from Nε His408 to O of CI in deacylation.
